# White matter microstructure of the neural emotion regulation circuitry in mild traumatic brain injury

**DOI:** 10.1111/ejn.15199

**Published:** 2021-04-02

**Authors:** Harm Jan van der Horn, Namrata R. Mangina, Sandra E. Rakers, Jelmer G. Kok, Marieke E. Timmerman, Alexander Leemans, Jacoba M. Spikman, Joukje van der Naalt

**Affiliations:** ^1^ Department of Neurology University Medical Center Groningen Groningen the Netherlands; ^2^ Department of Psychometrics and Statistics University of Groningen Groningen the Netherlands; ^3^ Image Sciences Institute University Medical Center Utrecht Utrecht the Netherlands

**Keywords:** concussion, connectivity, diffusion MRI, symptoms, tractography

## Abstract

Emotion regulation is related to recovery after mild traumatic brain injury (mTBI). This longitudinal tractography study examined white matter tracts subserving emotion regulation across the spectrum of mTBI, with a focus on persistent symptoms. Four groups were examined: (a) symptomatic (*n* = 33) and (b) asymptomatic (*n* = 20) patients with uncomplicated mTBI (i.e., no lesions on computed tomography [CT]), (c) patients with CT‐lesions in the frontal areas (*n* = 14), and (d) healthy controls (HC) (*n* = 20). Diffusion and conventional MRI were performed approximately 1‐ and 3‐months post‐injury. Whole‐brain deterministic tractography followed by region of interest analyses was used to identify forceps minor (FM), uncinate fasciculus (UF), and cingulum bundle as tracts of interest. An adjusted version of the ExploreDTI Atlas Based Tractography method was used to obtain reliable tracts for every subject. Mean fractional anisotropy (FA), mean, radial and axial diffusivity (MD, RD, AD), and number of streamlines were studied per tract. Linear mixed models showed lower FA, and higher MD, and RD of the right UF in asymptomatic patients with uncomplicated mTBI relative to symptomatic patients and HC. Diffusion alterations were most pronounced in the group with frontal lesions on CT, particularly in the FM and UF; these effects increased over time. Within the group of patients with uncomplicated mTBI, there were no associations of diffusion measures with the number of symptoms nor with lesions on conventional MRI. In conclusion, mTBI can cause microstructural changes in emotion regulation tracts, however, no explanation was found for the presence of symptoms.

## Graphical abstract

This diffusion MRI study examined frontal tracts supporting emotion regulation in patients with mild traumatic brain injury (mTBI). Asymptomatic patients with uncomplicated (i.e., no lesions on computed tomography [CT]) mTBI showed changes in the microstructure of the right uncinate fasciculus (UF), whereas symptomatic patients did not. Microstructural changes were most pronounced in patients with frontal lesions on CT, particularly in the forceps minor and UF. In uncomplicated mTBI, tract microstructure was not related to the number of symptoms or lesions on conventional MRI.

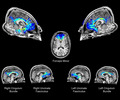

## 1 INTRODUCTION

Many patients with mild traumatic brain injury (mTBI) develop persistent symptoms, however, it is still difficult to explain these symptoms for the individual patient (Mayer et al., 2017; van der Horn, Out, et al., 2019; van der Naalt et al., 2017). Computed tomography (CT) and conventional MRI‐scans provide insufficient explanations in this respect (Jacobs et al., 2010; Karr et al., 2020; van der Horn et al., 2018; van der Naalt et al., 2017). Data suggest that recovery after mTBI is particularly influenced by someone's capacity to regulate negative emotions, which determines the ability to cope with the consequences of the injury (e.g., presence of symptoms, [temporary] changes in daily functioning) (van der Horn, Liemburg, et al., 2016; van der Naalt et al., 2017). The prefrontal cortex is the main area of the brain involved in the cognitive control of emotion, and this area is particularly vulnerable to TBI (Metting et al., 2009; Ochsner et al., 2012). The forceps minor (FM), uncinate fasciculus (UF), and cingulum bundle (CB) are key white matter tracts connecting frontal, limbic, and temporal areas within the emotion regulation circuitry (Ochsner & Gross, 2005; Versace et al., 2015). These tracts have found to be affected in mTBI (Aoki & Inokuchi, 2016; Dodd et al., 2014; Hellstrøm et al., 2017; Mayer et al., 2010; Wallace et al., 2020), and therefore, it can be hypothesized that microstructural injury to these tracts contributes to emotion regulation deficits, resulting in persistent symptoms. Investigating this matter may clear up some of the uncertainty regarding the association between structural brain lesions, especially within the frontal regions, and poor recovery after mTBI. However, so far, diffusion magnetic resonance imaging (dMRI) studies have paid little attention to this specific topic. In a previously published study on part of the current study sample, we did not find differences between symptomatic patients with uncomplicated mTBI (i.e., no lesions on day‐of‐injury CT [Williams et al., 1990]) and healthy controls (HC) with regard to graph measures of the total structural connectome (van der Horn, Kok, et al., 2016). However, it is plausible that specific lesions in frontal emotion regulation tracts might be related to symptoms, which can be missed using a graph‐theoretical approach. The current longitudinal dMRI tractography study zooms in on the microstructure of the FM, UF, and CB with the aim of finding explanations for persistent symptoms. An adjusted version of the ExploreDTI Atlas Based Tractography method was used to warp custom‐drawn regions of interest (ROI’s) from a template subject to all of the other subjects in order to dissect tracts for the individual subjects.

Patients across the mTBI severity spectrum were investigated. Two groups of patients with uncomplicated mTBI were included, a group with symptoms and a group without symptoms. In order to assess the influence of frontal macroscopic lesions on emotion regulation tract microstructure, we specifically selected a group of patients situated at the severe end of the mTBI spectrum that had frontal lesions on day‐of‐injury CT‐scans. In addition, a group of HC was included. Patients were measured at 1‐ and 3‐month post‐injury with the aim of finding possible recovery effects or increases in pathology as a function of time. For the group of patients with uncomplicated mTBI, it was also assessed whether there were relationships between diffusion measures and the number of symptoms, instead of dividing them into groups based on presence or absence of symptoms. Because patients with persistent symptoms often undergo conventional MRI (i.e., susceptibility‐weighted imaging [SWI] and T2*‐gradient echo [GRE]) at the outpatient clinic, we also added the presence/absence of lesions on conventional MRI as a variable of interest.

## 2 METHODS

### 2.1 Participants and clinical measures

As part of a prospective cohort study (UPFRONT‐study; March 2013‐February 2015) at a level I trauma center we studied four groups: 33 patients with uncomplicated mTBI with subjective post‐traumatic symptoms (PTS+), and 20 without symptoms (PTS−), 14 patients with complicated mild and moderate TBI (CT+) (with 5/14 moderate TBI), and 20 healthy controls (HC). Patients with uncomplicated mTBI were selected based on either the presence (≥3 symptoms; PTS+) or absence (<3) of symptoms (PTS−). Patients in the CT+ group were included regardless of symptom levels. PTS were measured at 2‐week post‐injury using the head injury symptom checklist and the presence of symptoms was defined as three or more (de Koning et al., 2016). Symptoms were measured again at 6‐month post‐injury. Radiologic details of lesions in the CT+ group are described in Table S1. The diagnosis mTBI was made according to the criteria of the American Congress of Rehabilitation Medicine (loss of consciousness [LOC] of max 30 min, after that a Glasgow Coma Scale [GCS] score of 13–15, post‐traumatic amnesia no longer than 24 hr) (Kayd et al., 1993). A diagnosis of moderate TBI was defined by LOC of more than 30 min, GCS 9–12, or PTA >24 hr (Einarsen et al., 2018; Godoy et al., 2016; Malec et al., 2007) Exclusion criteria were as follows: neurologic or psychiatric comorbidity, hospital admission for previous TBI, drug or alcohol abuse, insufficient comprehension of the Dutch language, intellectual disability, and contraindications for MRI. HC did not have a history of TBI, and this group was matched with the total TBI‐group regarding age, sex, education, and handedness. The study was approved by the local Medical Ethical Committee of the University Medical Center Groningen (METc), all patients provided written consent, and all procedures were carried out in compliance with the declaration of Helsinki.

Functional outcome was measured at 6‐months post‐injury using the Glasgow Outcome Scale Extended (GOS‐E), which is an 8‐point scale ranging from 8 (upper good recovery) to 1 (death) (Jennett et al., 1981).

### 2.2 Imaging acquisition and processing

Acquisition and processing were discussed in detail in previously published dMRI research (van der Horn, Kok, et al., 2016). For the sake of brevity, we will provide a brief overview of the conducted steps. Patients underwent longitudinal 3T MRI‐scanning at approximately 4 weeks, and 3‐month post‐injury (29/33 PTS+, 18/20 PTS−, and all CT+ returned for follow‐up scanning); HC were scanned only once. The following images were acquired: T1‐weighted, and dMRI (60 directions, *b* = 1,000 s/mm^2^, seven volumes with *b* = 0 s/mm^2^ averaged in one volume by the scanner), axial SWI, and coronal T2*‐GRE. In the uncomplicated mTBI group, 15 of 33 patients showed micro‐hemorrhagic lesions on SWI and/or T2*‐GRE; details regarding these groups can be found in (van der Horn et al., 2018). T1‐data were analyzed using the main FreeSurfer pipeline (Dale et al., 1999). Processing of dMRI data was performed using *ExploreDTI* version 4.8.5, which included correction for motion, eddy currents, and susceptibility distortions, followed by whole‐brain constrained spherical devolution (CSD) deterministic tractography (Leemans et al., 2009). Seed points were defined on a uniform rectilinear grid with a resolution of 2 × 2 × 2 mm^3^. The other tract parameters and stopping criteria used were as follows: step size of 1 mm, FOD threshold of 0.1, and angle threshold of 30°. A single tissue fiber response function was used with recursive calibration (Tax et al., 2014). No maximum number of whole‐brain streamlines was defined during tracking, and there was no SIFT/SIFT2 reduction applied. Diffusion results were quality checked for every subject by overlaying the fractional anisotropy (FA)‐maps on the T1‐image and viewing them for all planes using movie loops. For each subject, the T1‐file from the result of the FreeSurfer pipeline, the DWI data after corrections, and the whole brain streamline set were stored and used in the analyses below.

### 2.3 Tract reconstruction

In order to dissect the FM, UF, and CB in each subject, we used an approach similar to the atlas‐based tractography that is available with ExploreDTI (Lebel et al., 2008). First, a template‐subject was chosen from the set of HC. Then, for each subject, a registration between the template's T1 volume and the subject's T1 volume was performed using Elastix version 4.7 (Klein et al., 2010; Shamonin et al., 2014). The moving image was the template's T1 volume and the fixed image the subject's T1 volume. We used a mask for the fixed image to restrict and therefore enhance the registration. This mask was a binary map, based on a dilated FA map of the subject. We used parameters as stored in the Elastix Parameter file database (http://elastix.bigr.nl/wiki/index.php/Parameter_file_database), par0000, which contains two parameter files (these proved to be most reliable after manually checking the results of some of the stored T1 registering parameter files). The resulting calculated transformation parameters (i.e., the transformation parameters that could be used to warp the template to each and every subject) were stored for use in subsequent analyses.

For each bundle, in the template subject AND and NOT ROIs were drawn in ExploreDTI in order to dissect the bundle from the whole brain streamline set. Drawing an ROI in ExploreDTI results in an ROI that is 1 voxel thick. Because later these ROIs would be warped non‐linearly between template and subjects, leaving the ROIs this way (one voxel thick), tearing/shearing the ROI would result in holes within the ROI, which would lessen the number of streamlines that should be retained later (for AND ROIs) or lessen the number of streamlines that should be excluded (for NOT ROIs), and thus would result in less reliable dissection results. Therefore, the ROIs drawn in the template subject were dilated to make them 3 voxels thick. For example, when an ROI was drawn in *X*‐plane 36, the *Y* and *Z* coordinates of all voxels that were included in this ROI, were 'copied' to *X*‐plane 35 and *X*‐plane 37, constructing a ROI that was 3 layers of voxel thick. That way, we were sufficiently sure no holes would appear when applying registrations between two brains, while still retaining essentially all of the ROI’s specificity.

The ROIs were warped using Transformix version 4.7 using the transformation parameters calculated by Elastix before, which resulted in the ROIs for a specific bundle warped to each and every subject (Klein et al., 2010; Shamonin et al., 2014). Then, the whole‐brain streamline sets of all subjects were restricted according to these warped ROIs.

**FIGURE 1:**
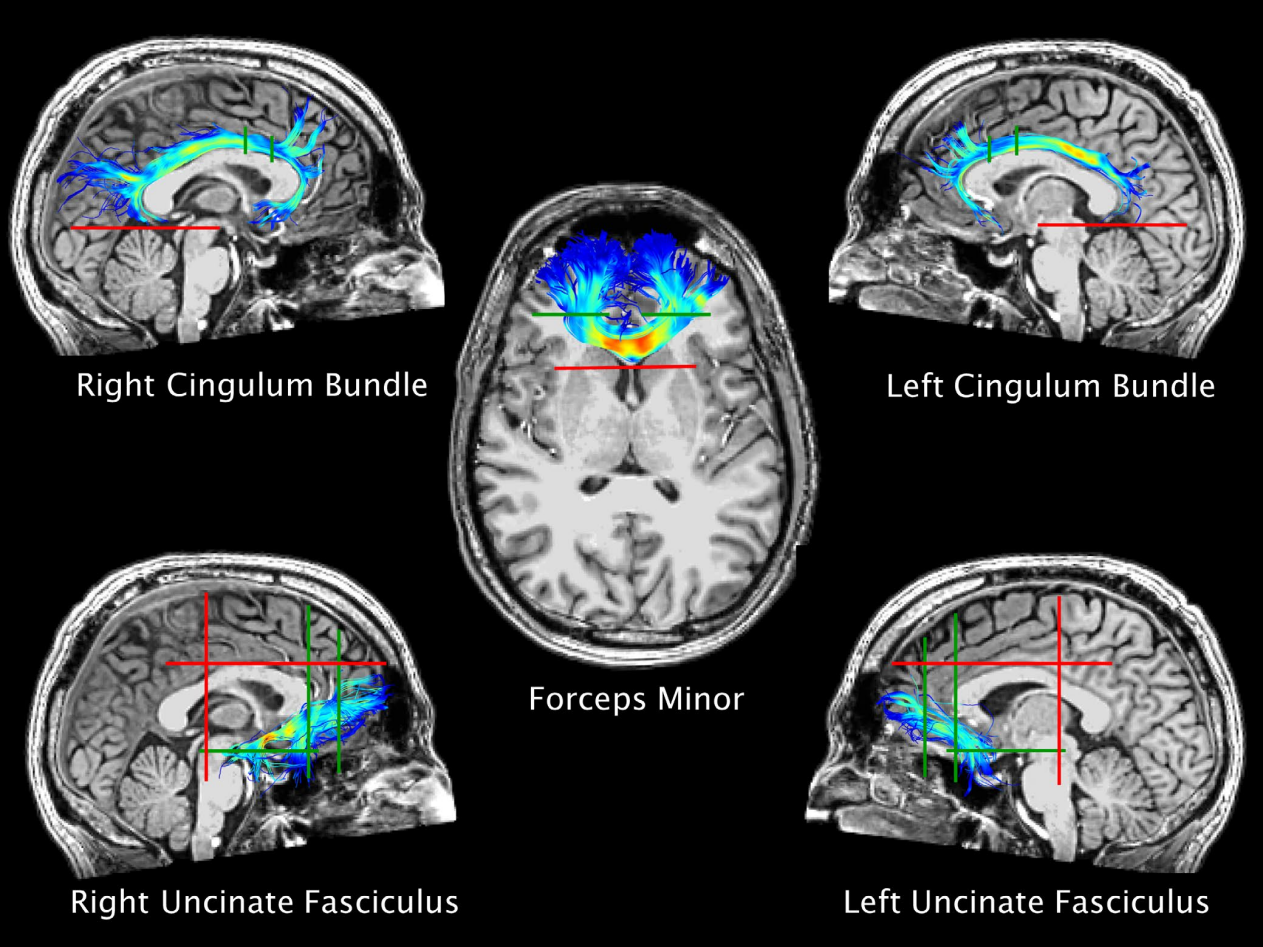
Tracts of interest in the template subject. Green lines show main AND‐ROIs, red lines show main NOT‐ROIs

Because satisfactory results were not expected after the first run, we iteratively enhanced the ROIs by starting with applying the aforementioned method to a subset of 20 subjects. After visually inspecting screenshots of the results, we adjusted the arrangement of AND and NOT ROIs in the template subject, repeated the analyses and again viewed the results, and so on, until the researchers (H.J.v.d.H. and N.R.M.) were satisfied that the results showed consistency with the known anatomical configuration of the bundles. Then, the ROIs were warped to all other subjects. Screenshots of the tracts of all the subjects were also briefly inspected for gross abnormalities after warping. Figure 1 shows the tracts of interest drawn in the template‐subject, and the locations of the main ROIs. A movie of the tracts of interest was added as Supporting Information 2.

Placement of ROIs for FM, UF, and CB was derived from previously published methods, and adjusted when deemed necessary (Catani & Thiebaut de Schotten, 2008; Coad et al., 2017; Folloni et al., 2019; Jones et al., 2013; Wakana et al., 2007). For FM two main coronal AND‐ROIs were placed in both hemispheres just anterior to the genu. A coronal NOT‐ROI was drawn just posterior to the genu of the corpus callosum. Additional NOT‐ROIs were put into place to exclude fibers that were inconsistent with the known anatomy of the FM.

Left and right UF were reconstructed separately, and main ROIs were kept consistent for left and right. Two coronal AND‐ROIs were placed: one just anterior to the rostral section of the genu of the corpus callosum, capturing the white matter tracts running in the anterior‐to‐posterior direction, and one at the border of the frontal and temporal lobe encompassing all of the white matter running from the frontal to temporal and limbic regions. One axial AND‐ROI was drawn on a slice at the height of the middle part of the mesencephalon, capturing the fibers running in the rostral‐caudal direction. The most optimal results were obtained with the AND‐ROIs covering the entire hemisphere within the respective planes, and adding NOT‐ROIs to remove inconsistent fibers. This resulted in the inclusion of amygdalofugal fibers and more longitudinal (non‐hook shaped) frontolimbic and frontotemporal fibers; for the sake of readability, we will refer to the entire tract as UF (Ebeling & Cramon, 1992; Folloni et al., 2019). Two primary NOT‐ROIs were drawn: one covering the entire hemi‐coronal plane at the level of the posterior part of the pons to ensure exclusion of the inferior fronto‐occipital fasciculus, and one in the axial plane just superior to the upper part of the body of the corpus callosum to exclude fibers running in the rostro‐caudal direction (e.g., the corona radiata). Additional NOT‐ROIs were placed to exclude tracts that were inconsistent with the known anatomy of the UF (e.g., one in the sagittal plane between the hemispheres to exclude fibers of the anterior commissure, which are known to intermingle with fibers of UF [Ebeling & Cramon, 1992]).

Left and right CB were also reconstructed separately, with similar main ROIs for both sides. As we were primarily interested in the frontal (mostly subgenual) tracts of CB we placed one coronal AND‐ROI just anterior to the middle of the body of the corpus callosum, and one coronal AND‐ROI anterior to the first ROI, in line with the back of the curve of the genu of the corpus callosum. The rationale for the latter (and not for placing the second ROI as was done in [Jones et al., 2013]) was the absence of fiber tracts that curved all the way around the genu in a few subjects, and an evidently lower number of tracts in general. To exclude the hippocampal part of the CB, an axial NOT‐ROI was placed, covering both hemispheres, in line with the upper part of the cerebellum. Additional NOT ROIs were placed to exclude tracts that were inconsistent with the known anatomy of the CB (e.g., one in the coronal plane to exclude fibers of the fornix).

**FIGURE 2:**
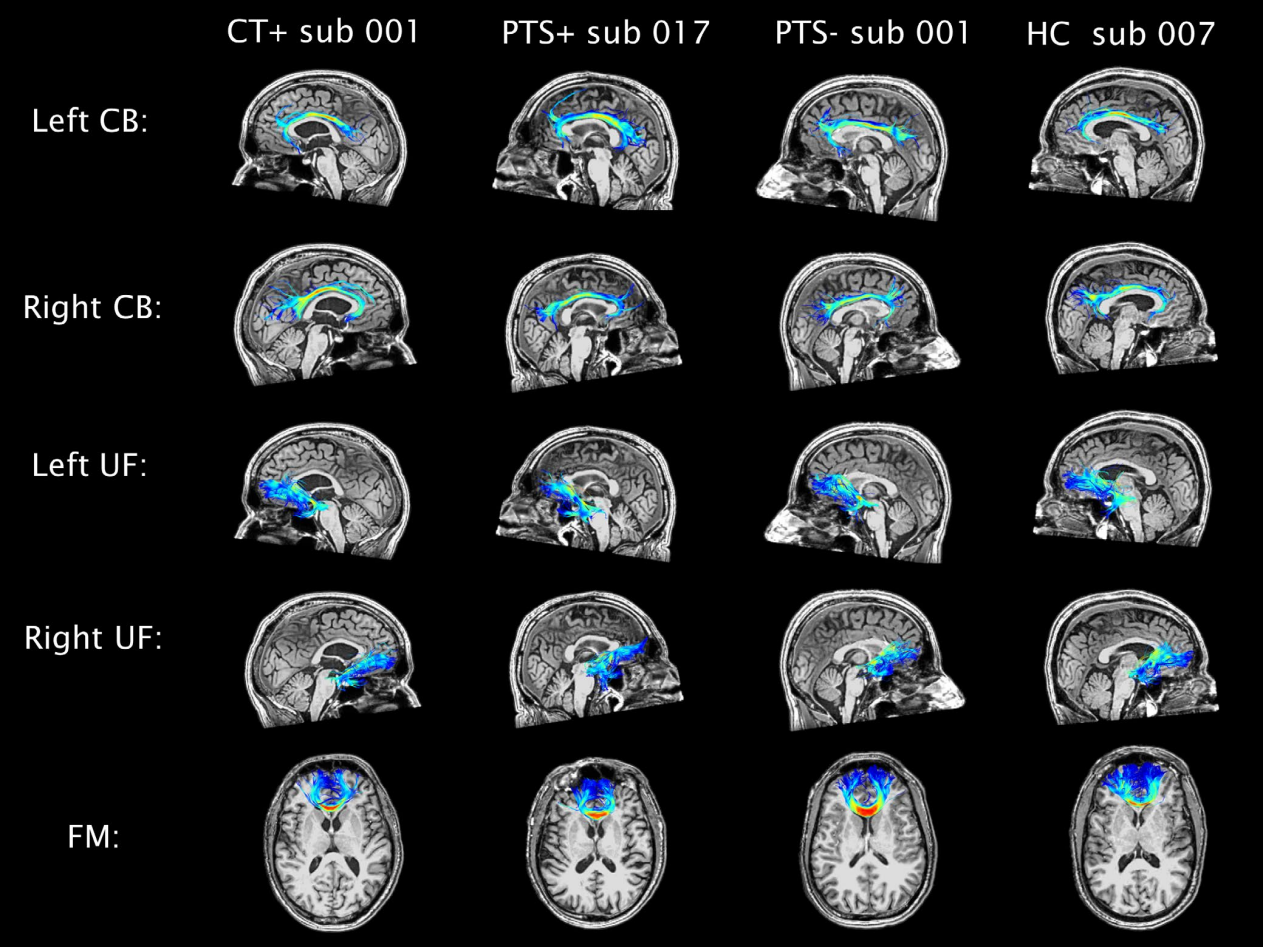
Tracts of interest after warping for four random subjects (one out of each subgroup). CB, cingulum bundle; CT+, group with frontal CT lesions; FM, forceps minor; HC, healthy controls; PTS−, group without post‐traumatic symptoms; PTS+, group with post‐traumatic symptoms; UF, uncinate fasciculus

Figure 2 shows the tracts after warping to a random subject out of every study group. Mean FA, MD, RD, AD, and number of streamlines per tract were stored for subsequent statistical analyses.

### 2.4 Statistics

Statistical testing of demographic, clinical, and neuropsychological data was performed using the Statistical Product and Service Solutions (SPSS version 20; IBM Corp.). For non‐normally distributed continuous, and for ordinal variables, Kruskall–Wallis and Wilcoxon rank‐sum tests were used. For nominal variables, Chi‐square tests were used.

Statistical testing of group and time effects per diffusion measure was performed using R Statistical Software (R Core Team, 2020) with a generalized linear mixed effects model (*glmer* function in the LME4 package: https://cran.r‐project.org/web/packages/lme4/ [Bates et al., 2015]). Group (i.e., PTS+, PTS−, CT+, HC) and time (4 weeks, and 3‐month post‐injury) were dummy coded, with HC and 4 weeks as the reference categories. Age, sex (dummy coded), and education (dummy coded) were included as covariates, because of their known relationship with white matter microstructure (Dunst et al., 2014; Gunning‐Dixon et al., 2009; Hsu et al., 2008; Kanaan et al., 2012). The following linear mixed model formula was used:

$$\begin{array}{c} Y∼1+G1+G2+G3+G1:\text{Time}+G2:\text{Time}+G3:\text{Time}+\\ \text{Age}+\text{Sex}+\text{Education}+(1|\text{Subject}) \end{array}$$

Groups (G) 1, 2, and 3 refer to PTS+, PTS−, and CT+, and HC were used as reference. Model fit was evaluated by inspecting the fitted versus original values (y), inspecting the variation and distribution of the residuals, computing whether or not the residuals were normally distributed using (one sample) Kolmogorov–Smirnov tests, and adjusting the family in the model (Gaussian, inverse Gaussian, binomial, gamma, etc.) when necessary. Post hoc tests to compare groups, and to compare time effects between groups (in case more than one group showed a significant time effect) were performed using the *glht* function in the “multcomp” package (https://cran.r‐project.org/web/packages/multcomp/index.html [Hothorn et al., 2008]). Alpha was set at 0.05, and false discovery rate corrections were applied to post hoc comparisons (Benjamini & Hochberg, 1995).

Additional mixed model analyses were performed per diffusion measure to examine the effect of the number of symptoms at 2 weeks, and the presence (*n* = 15) or absence (*n* = 38) of lesions on conventional MRI (SWI and T2‐GRE). For these analyses the following model formula was used:

$$\begin{array}{c} Y∼1+\text{Symptoms}\times \text{Time}+\text{Lesions}\times \text{Time}+\text{Lesions}\times \\ \text{Symptoms}+\text{Age}+\text{Sex}+\text{Education}+(1|\text{Subject}) \end{array}$$

## 3 RESULTS

### 3.1 Participant characteristics

Table 1 depicts the demographical, and clinical characteristics. There were significantly fewer female patients in the PTS− group as compared to PTS+ (*χ* ^2^ = 8.22, *p* < 0.01); no significant differences in sex were present between any of the patient subgroups and HC. Relative to PTS− (*χ* ^2^ = 12.49, *p* = 0.01) and PTS+ (*χ* ^2^ = 9.30, *p* = 0.05), CT+had a lower GCS score; there was no significant difference between PTS− and PTS+. Compared to PTS+ (*χ* ^2^ = 11.57, *p* < 0.01) and CT+ (*χ* ^2^ = 8.47, *p* = 0.01), PTS− had a significantly higher GOS‐E score at 6‐month post‐injury. For PTS+and CT+, GOS‐E scores were similar. At both time points, there was no significant difference in the number of symptoms between PTS+and CT+. There was a significantly lower number of symptoms in PTS− compared to CT+ at both 2 weeks (*W* = 210, *p* < 0.01), and 6 months (*W* = 220.5, *p* < 0.01) post‐injury. Uncomplicated mTBI patients with and without lesions on conventional MRI reported a similar number of symptoms at 2 weeks (median = 7 vs. 8, respectively; *W* = 260.5, *p* = 0.63) and 6 months (median = 4.5 vs. 5; *W* = 154, *p* = 0.57) post‐injury. None of the patients reported pre‐injury mental disorders (NB: there were two missing values for the CT+ group). For the group of six patients that did not return for follow‐up scanning, the mean age was 39 years, 33% was female, and the median education level was 5; these data are comparable to that of the patients that stayed in the study.

**TABLE 1:** Participant characteristics

	CT+ (*n* = 14)	PTS+ (*n* = 33)	PTS− (*n* = 20)	HC (*n* = 20)	*p*‐value; test‐statistic
Age, years, median (range)	44.5 (19–59)	33 (19–63)	34 (20–64)	29.5 (18–61)	0.81; *H* = 0.98
Sex, % female	21.4	48.5	10	30	0.02; *χ* ^2^ = 9.44
Education level, median (range)^a^	5.5 (5–7)	6 (4–7)	6 (2–7)	6 (5–7)	0.49; *χ* ^2^ = 11.49
GCS‐score, median (range)	14 (9–15)	14 (13–15)	15 (13–15)	N/A	0.01; *χ* ^2^ = 20.51
Days between injury & 1st visit, median (range)	40.5 (29–67)	32 (22–56)	32.5 (22–69)	N/A	0.06; *H* = 5.61
Days between injury & 2nd visit, median (range)	94 (85–127)	92 (61–207)	94 (77–126)	N/A	0.46; *H* = 1.55
LOC					0.37; *χ* ^2^ = 4.26
No	14.3	27.3	10	N/A	
<15 min (% of patients)	71.4	69.7	80	N/A	
>15 min (% of patients)	14.3	3	10	N/A	
PTA (yes; % of patients)	100 (*n* = 14)	90.9 (*n* = 33)	73.7 (*n* = 19)	N/A	0.06; *χ* ^2^ = 5.81
GOS‐E, median (range)	8 (6–8) (*n* = 11)	7 (5–8) (*n* = 23)	8 (7–8) (*n* = 19)	N/A	0.03; *χ* ^2^ = 14.22
Number of symptoms at 2 weeks, median (range)	9 (2–17) (*n* = 11)	9 (5–16) (*n* = 33)	0 (0–1) (*n* = 20)	N/A	<0.001; *H* = 42.09
Number of symptoms at 6 months, median (range)	7 (0–14) (*n* = 10)	7 (0–18) (*n* = 26)	0 (0–5) (*n* = 18)	N/A	<0.001; *H* = 28.08

Abbreviations: CR, complete recovery; GCS, Glasgow Coma Score; GOS‐E, Glasgow Outcome Scale Extended; HC, healthy controls; ICR, incomplete recovery; LOC, loss of consciousness; MRI, magnetic resonance imaging; N/A, not applicable; PTA, posttraumatic amnesia.

^a^ Education level was based on a Dutch classification system, according to Verhage (1964), ranging from 1 to 7 (highest).

### 3.2 Group effects, for patients at 4 weeks post‐injury

**FIGURE 3:**
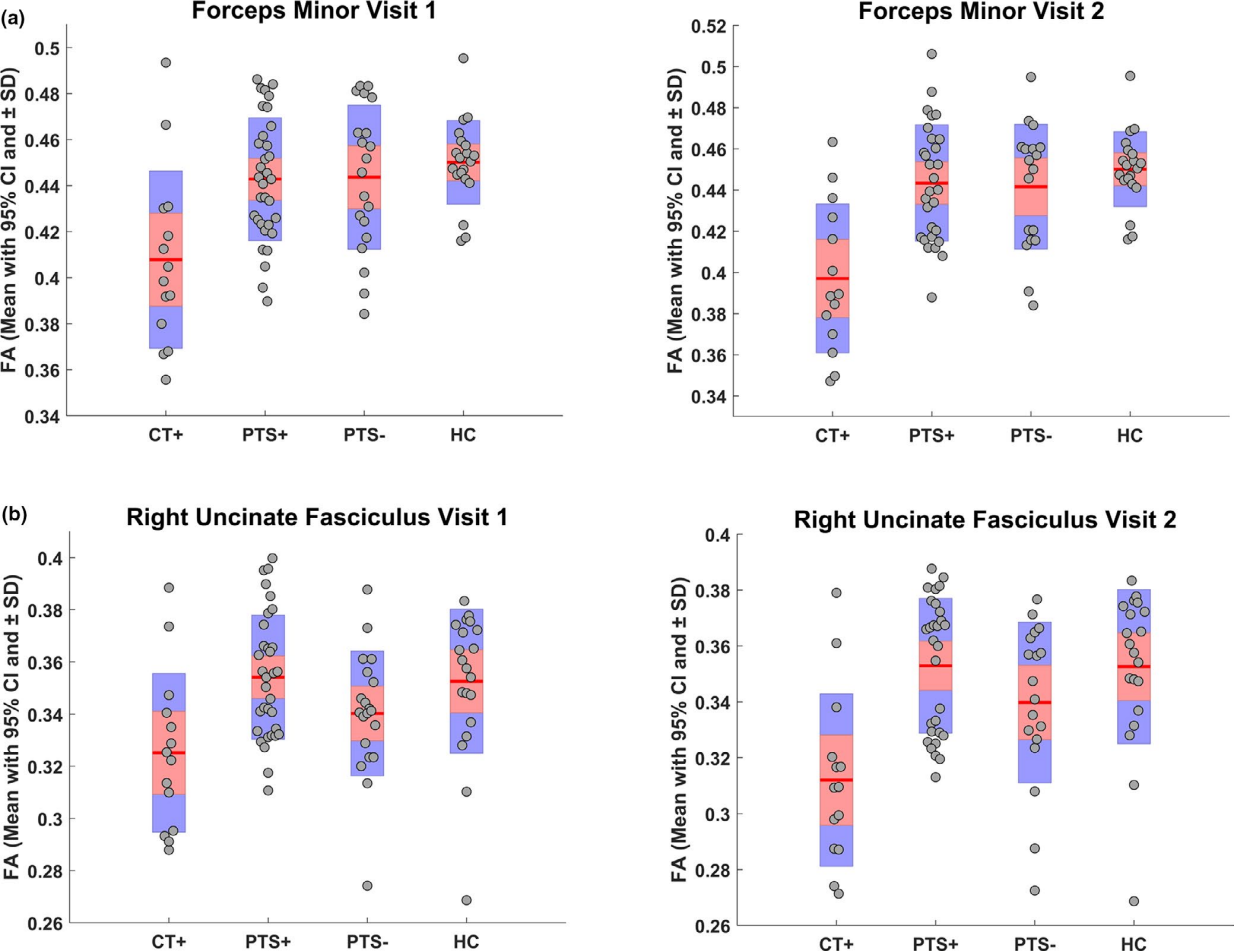
Mean fractional anisotropy (FA) for (a) forceps minor, and (b) right uncinate fasciculus. Confidence interval is shown in red; ±1 standard deviation is shown in blue. Plots were made using notBoxPlot (version 1.31) implemented in Matlab. CT+, group with frontal CT lesions; HC, healthy controls; PTS−, group without post‐traumatic symptoms; PTS+, group with post‐traumatic symptoms

Linear mixed models showed no significant differences at 4‐week post‐injury regarding mean FA of the FM between PTS+ and PTS−, nor between either of these subgroups and HC. However, a significantly lower mean FA was found in CT+ relative to HC (*p* < 0.01), PTS+ (*p* < 0.01), and PTS− (*p* < 0.01) (Figure 3a). Similar effects in the opposite direction were observed for mean MD (all *p* < 0.05), and RD (all *p* < 0.01). The number of streamlines for FM was lower in CT+ and PTS+ relative to HC (both *p* = 0.01). No significant group effects were found for mean AD.

For left UF there was only a significantly higher mean RD in CT+ versus PTS+ (*p* = 0.04). No further group differences were found.

For right UF there was a significantly lower mean FA in PTS− relative to HC (*p* = 0.04) and PTS+ (*p* < 0.01) (Figure 3b). For the PTS− group, mean MD was higher relative to the PTS+ (*p =* 0.03), and mean RD was higher relative to the PTS+ (*p* < 0.01) and HC group (*p* = 0.03). A significantly lower mean FA was found in CT+ relative to HC (*p* < 0.01), and PTS+ (*p* < 0.01). Also, a significantly higher MD and RD value was found for CT+ versus HC (both *p* ≤ 0.01), and PTS + (both *p* < 0.01). No significant effects were observed for mean AD and number of streamlines.

No significant group effects were present regarding any of the diffusion measures for the left nor right CB.

### 3.3 Longitudinal effects

Within the PTS+, and PTS− groups, diffusion measures did not significantly change from 4‐week to 3‐month post‐injury. Within the CT+group, mean FA values for the FM decreased significantly over time (*p* < 0.01) (see Figure 3a for mean FA at both time points). An opposite effect was observed for mean MD, RD, and AD of FM (all *p* < 0.01). For mean MD, RD, and AD (all *p* < 0.01), but not for mean FA, of the right UF, similar effects were observed in the CT+ group.

### 3.4 Number of symptoms and lesions on conventional MRI

**FIGURE 4:**
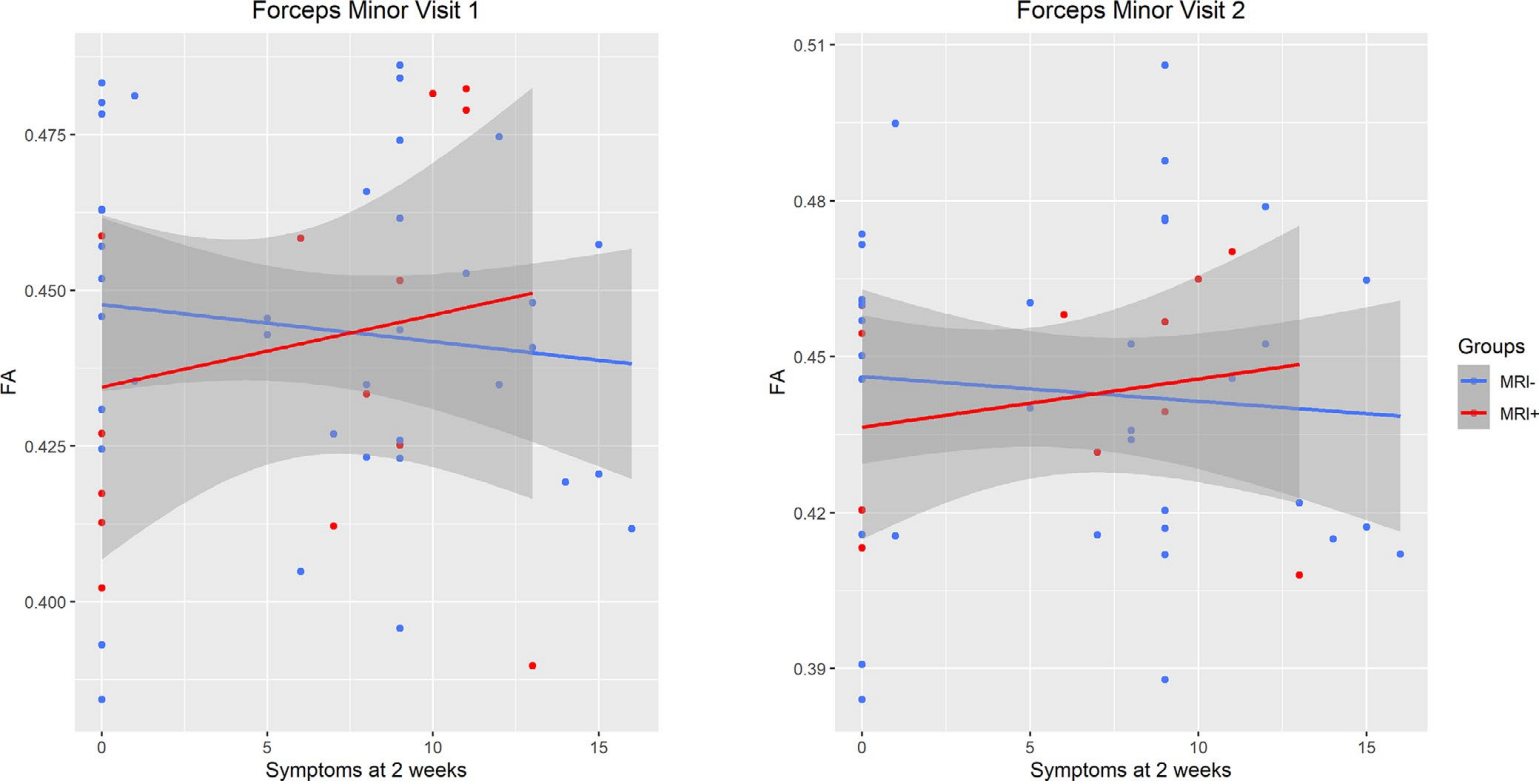
Interaction between number of symptoms at 2 weeks post‐injury and presence/absence of lesions on conventional MRI for mean fractional anisotropy (FA) of the forceps minor at both time points in the uncomplicated mTBI group. Confidence interval (95%) is shown around trend line. Plots were made using ggplot2 (version 3.3.3) functions implemented in R

Regarding diffusion measures in the group with uncomplicated mTBI, no significant effects were found for the number of symptoms, nor for the presence/absence of lesions on conventional MRI, nor for the interaction between these two variables. Figure 4 illustrates the non‐significant correlation between mean FA of FM and the number of symptoms for the groups with and without lesions during both visits.

## 4 DISCUSSION

This study indicates that microstructural injury to the neural emotion regulation circuitry is not likely to play a role in causing poor recovery after uncomplicated mTBI. Interestingly, it was the asymptomatic, and not the symptomatic group of patients with uncomplicated mTBI, that showed changes in the microstructure of the right UF. As expected, microstructural white matter injury was most pronounced in patients with macroscopic frontal lesions, as detected with CT‐scans, and this was most evident in the FM and right UF. Within this group, abnormalities in diffusion measures were found to increase over time, suggesting ongoing pathological processes for months after injury. Lastly, in the group with uncomplicated mTBI, there were no relationships of tract microstructure with symptoms levels, nor with lesions on conventional MRI (i.e., T2‐GRE, SWI).

The frontal lobe is vulnerable to TBI (Bigler, 2007). Since the frontal lobe contains areas that are important for regulating negative emotions, it is plausible that injury to the connecting white matter tracts might cause problems with psychological adaptation to symptoms and changes in daily functioning after mTBI (van der Horn, Out, et al., 2019). To investigate microstructural connectivity within the frontal emotion regulation circuitry across the entire spectrum of mTBI, we carefully selected patients with uncomplicated mTBI who did and did not report symptoms, as well as a group of patients with macroscopic lesions in the frontal areas, as measured with CT. The uncomplicated group with symptoms did not show diffusion abnormalities relative to HC, at 1 nor at 3‐month post‐injury, which leads us to believe that microstructural injury to frontal emotion regulation tracts is not related to the development of PTS. Remarkably, in the group without symptoms, the microstructure of the right UF was changed as compared to the group with symptoms, and to a lesser extent also compared to HC, although these effects were not as extensive as for the group with frontal CT lesions. These findings provide further support for the hypothesis that the presence of symptoms is not related to micro‐structural injury in emotion regulation tracts, but to pre‐existent psychological characteristics instead, as was suggested by our previous research (van der Horn, Out, et al., 2019). Although highly speculative, it is tantalizing to elaborate on a possible protective effect of compromised UF microstructure regarding the development of persistent symptoms via the presence of less emotional awareness. In line with our results, previous diffusion and resting‐state fMRI studies have shown null findings with respect to persistent symptoms in pediatric and adult mTBI (Ilvesmaki et al., 2016; Stephenson et al., 2020; Wäljas et al., 2014). Other studies have reported diffusion abnormalities in the same tracts that were examined in our study, and that these abnormalities were related to PTS (Aoki & Inokuchi, 2016; Dodd et al., 2014; Hellstrøm et al., 2017; King et al., 2019; Mayer et al., 2010; Wallace et al., 2020). However, these studies did not specifically focus on emotion regulation tracts (but on a wider variety of tracts), did not all include analyses of symptoms, and used different methodology (i.e., no tractography). A recent study has demonstrated that even though diffusion abnormalities within the frontal part of the corpus callosum could be detected at 6 months after sports‐related mTBI, abnormalities within this region were associated with symptoms only in the acute phase (24–48 hr) (Wu et al., 2020). One has to realize that the group that was examined in this study was asymptomatic within 2‐to 3‐week post‐injury, which means this group was located at the milder end of the mTBI spectrum. Nevertheless, in our study, we might have missed abnormalities that could have been present at an earlier timeframe, even in the asymptomatic group.

As expected, patients with macroscopic frontal lesions on CT, also showed diffusion changes in frontal tracts. These changes seem to become more pronounced as a function of time, which indicates that pathological processes underlying white matter injury have a protracted course. Although, the comparisons with both uncomplicated patients and HC indicate that these changes (e.g., FA decrease) are related to microstructural injury, it has to be realized that both directions of diffusion changes (i.e., FA increases and decreases) could indicate pathology, and that also methodological factors, such as number of diffusion directions, are of influence (Dodd et al., 2014). Patients within the group with CT‐lesions reported similar numbers of symptoms as the uncomplicated group with symptoms, and they did not have a poorer functional outcome (measured with the GOS‐E). Cohort studies have demonstrated that inclusion of CT‐characteristics in statistical models does not result in better prediction of outcome after mTBI, which fits with our results (Jacobs et al., 2010; van der Naalt et al., 2017). Furthermore, it has been shown that lesions on CT are not related to symptoms, nor to neuropsychological measures in patients with mTBI (Iverson et al., 2019; Karr et al., 2020). In the current study, we also did not find any relationships between diffusion measures, symptom levels, and lesions on conventional MRI in the group with uncomplicated mTBI. Interestingly, a recent study has also shown that patients without lesions on CT or MRI had a higher number of acute symptoms compared to those with lesions, despite lower levels inflammatory markers, which further questions the relevance of performing structural MRI in patients with persistent symptoms (Edwards et al., 2020). Altogether, these findings underscore the complexity of the causative mechanism of persistent symptoms. It would be exciting to see future larger‐scale cohort studies incorporating longitudinal diffusion measures of emotion regulation tracts as well as conventional imaging, biochemical data, and psychometric parameters of emotion regulation in models for the prediction of persistent symptoms and poor outcome after mTBI.

There are several strengths of this study. First, we specifically included a group of patients with CT‐lesions in the frontal areas to examine the influence of macroscopic frontal lesions on the microstructure of tracts involved in emotion regulation. The prefrontal cortex plays a pivotal role in the cognitive control of emotion, however, little is known about the association of frontal lesions on CT with recovery at the milder end of the TBI spectrum (Ochsner et al., 2012). Therefore, we consider the inclusion of patients with CT lesions confined to the frontal areas as a strength of our study, although we acknowledge the fact that it is a small group and thus further research is warranted. An additional benefit of including this group is that it indirectly served as a quality assurance measure, as it would be expected to find microstructural lesions in this group. One could argue that inclusion of patients with moderate TBI might obscure the results related to mild TBI. However, these moderate TBI patients were located near the severe end of the mild TBI spectrum based on clinical characteristics (van der Horn, et al., 2020). Noticeably, microstructural injury of several mTBI patients in this group was comparable to patients with moderate TBI, underlining the fact that injury severity is a continuum, which is difficult to categorize using clinical characteristics (Malec et al., 2007; Teasdale et al., 2014). A recent opinion paper has recommended abandoning current severity labels, and adopting risk assessments using multimodal data instead (Tenovuo et al., 2021). Altogether, we consider it a strength of our study that a broad range of patients with mTBI were included, with moderate TBI and HC at the borders of the spectrum. A second strength of our study is that we collected longitudinal diffusion MRI data, which enabled us to examine whether or not abnormalities in diffusion measures were transient, or increasing with time. Third, we used CSD‐tractography, which results in fewer false negative tracts as compared to diffusion tensor imaging (Tournier et al., 2007). Lastly, we used an adjusted version of the ExploreDTI Atlas Based Tractography method, where improved registration, thicker ROIs (resulting in warped ROIs without holes) and conveniently checking results using screenshots resulted in the reliable dissection of the tracts of interest.

There are also (additional) limitations that need to be addressed. First, there were some missing values for clinical follow‐up measurements. Second, the *b*‐value used in our study (1,000 s/mm^2^) is relatively low for CSD‐tractography, which may have influenced the identification of crossing fibers. Third, we chose to look at the mean FA/MD/RD/AD of the resulting streamline bundles, where, for example, true differences in the mean FA at a specific location in a bundle may have been obscured by the (variation of) normal FA values of the “normal” part of the bundle. A solution here could be to perform along‐tract analysis (which does have its own shortcomings). Fourth, we did not administer any emotion regulation tasks or questionnaires, which impedes us from drawing conclusions regarding relationships with actual emotion regulation processes. Fifth, our study sample size is relatively small, which impedes drawing definitive conclusions about the role of microstructural injury to emotion regulation tracts in the development of persistent symptoms. Finally, there is some delay between filling in 2‐week questionnaires and first scans (due to time related to inclusion, and scheduling). We acknowledge that it would have been more appropriate to also measure symptoms at time of scanning. However, we are certain that the symptom status did not change during this interval, since almost all of the patients with symptoms were still symptomatic at 6‐month post‐injury.

To summarize, we have shown that the presence of post‐traumatic symptoms after uncomplicated mTBI was not related to microstructural injury to the neural emotion regulation circuitry. Furthermore, within this group of patients, there were no effects of number of symptoms or conventional MRI results on tract diffusion measures. In patients with frontal lesions on CT, pathological effects were found in several tracts, that worsened over time, although these effects did not translate into poor clinical recovery. An interesting avenue for future research might be to investigate whether or not there is a link between psychological, biochemical, and microstructural white matter parameters that explains the persistence of symptoms after mTBI.

## CONFLICTS OF INTEREST

None of the authors report any conflicts of interest.

## AUTHORS’ CONTRIBUTIONS

J.v.d.N. and J.M.S. designed and supervised the study. H.J.v.d.H. collected the data. He also analyzed the data, with help from J.G.K., N.R.M., S.E.R., A.L., and M.E.T., and drafted/revised the manuscript. All authors provided feedback on manuscript drafts.

## ETHICAL STATEMENT

The study was approved by the local Medical Ethical Committee (METc) of the University Medical Center Groningen, The Netherlands. All participants gave written informed consent. All procedures were carried out in accordance with the declaration of Helsinki.

### PEER REVIEW

The peer review history for this article is available at https://publons.com/publon/10.1111/ejn.15199.

## Supporting information

Table S1

Video S1

## Data Availability

Data are available for other researchers upon reasonable request.
