# Supplementary material for: White matter microstructure of the neural emotion regulation circuitry in mild traumatic brain injury
Source: Eur J Neurosci. 2021 Apr 2;53(10):3463–75. doi: 10.1111/ejn.15199 (PMC8251942; doi:10.1111/ejn.15199)
Supplement: Supplementary file 1 — Table S1 [file EJN-53-3463-s001.docx]

**Supplementary Table 1:** Characteristics of CT-lesions.

|  | **Marshall Score** | | **Midline Shift** | **Contusion (volume in cm^3^)** | **SDH/EDH/SAH** | **Micro-hemorrhages** |
| --- | --- | --- | --- | --- | --- | --- |
| *Patient* |  | |  |  |  |  |
| 1 | 2 | no | | frontal left (0.1) | SAH |  |
| 2* | 2 | no | | frontal left (0.34) | SAH | yes, cortical |
| 3 | 5 | 6 mm | | frontal right (60) | EDH right |  |
| 4 | 2 | no | | frontal left & right (2 & 4) | EDH right |  |
| 5 | 2 | no | |  | EDH right frontal |  |
| 6 | 2 | no | | frontal left (0.05) |  |  |
| 7 | 2 | no | | frontal right (0.11) | SAH |  |
| 8 | 2 | no | | frontal right (0.9) | SDH right |  |
| 9 | 2 | no | | frontal left (20.1) |  |  |
| 10* | 2 | no | | frontal left (0.19) |  | yes, cortical |
| 11* | 2 | no | | frontal left & right (8.4 & 12.6) | SAH |  |
| 12 | 2 | no | |  | SDH bilat frontal/SAH |  |
| 13* | 2 | no | | frontal left>right (11.3) | SDH bilat/EDH left/SAH |  |
| 14* | 2 | no | | frontal right (3.1) | EDH left/SDH right |  |
| *patients with moderate TBI. | | | | |  |  |

*EDH = epidural hematoma, SAH = subarachnoid hemorrhage, SDH = subdural hematoma.*
